# Prognosis of non-small-cell lung cancer in patients with idiopathic pulmonary fibrosis

**DOI:** 10.1038/s41598-019-49026-y

**Published:** 2019-08-29

**Authors:** SongYi Han, Yeon Joo Lee, Jong Sun Park, Young-Jae Cho, Ho Il Yoon, Jae-Ho Lee, Choon-Taek Lee, Jin-Haeng Chung, Kyung Won Lee, Sang Hoon Lee

**Affiliations:** 1 Division of Pulmonary and Critical Care Medicine, Department of Internal Medicine, Seoul National University Bundang Hospital, 82 Gumi-ro, 173 Beon-gil, Bundang-gu, Seongnam-si, Gyeonggi-do 463-707 Republic of Korea; 2 Division of Hospital Medicine, Department of Internal Medicine, Yonsei University College of Medicine, Yonsei University Health System 50-1 Yonsei-ro, Seodaemun-gu, Seoul 120-752 Korea; 3 Department of Pathology, Seoul National University Bundang Hospital, 82 Gumi-ro, 173 Beon-gil, Bundang-gu, Seongnam-si, Gyeonggi-do 463-707 Republic of Korea; 4 Department of Radiology, Seoul National University Bundang Hospital, 82 Gumi-ro, 173 Beon-gil, Bundang-gu, Seongnam-si, Gyeonggi-do 463-707 Republic of Korea; 5 Division of Pulmonary and Critical Care Medicine, Department of Internal Medicine, Institute of Chest Diseases, Severance Hospital, Yonsei University College of Medicine. 50-1 Yonsei-ro, Seodaemun-gu, Seoul 120-752 Korea

**Keywords:** Outcomes research, Non-small-cell lung cancer

## Abstract

The risk of lung cancer is higher in idiopathic pulmonary fibrosis (IPF) because both conditions share common risk factors. However, no standard treatment modality for LC in IPF exists due to rare incidence, poor prognosis, and acute exacerbation (AE) of IPF during treatment. We aimed to determine the efficacy of LC treatments and the prognosis in LC patients with IPF according to the LC stage and GAP (gender [G], age [A], and two physiology variables [P]) stage. From 2003 to 2016, 160 retrospectively enrolled patients were classified according to the LC clinical stage and GAP stage. The average (±standard deviation) patient age was 70.1 ± 8.2 years; the cohort predominantly comprised men (94.4%). In GAP stage I, surgery was significantly associated with better survival outcomes in LC. In contrast, no treatment modality yielded significant clinical improvement in GAP stage II/III. The incidences of AE in IPF and its mortality during treatment were 13.8% and 6.3%, respectively. AE occurred commonly in advanced GAP stage. Active treatment should be considered in GAP stage I. The performance status and LC stage should be considered when deciding about the necessity of surgery for patients in advanced GAP stage.

## Introduction

Idiopathic pulmonary fibrosis (IPF) is the most frequent and severe type of idiopathic interstitial pneumonia (IIP) with an unknown aetiology. IPF has a median survival of approximately 2 to 3 years after diagnosis and presents with a histologic pattern of usual interstitial pneumonia (UIP) on computed tomography (CT)^1^. Disease progression is characterised by ongoing fibrosis, worsening dyspnoea, and decreasing pulmonary function tests (PFT), particularly forced vital capacity (FVC) and diffusing capacity for carbon monoxide (DL_CO_)^2,3^.

Emerging evidence shows that IPF is an important risk factor for lung cancer (LC) development^4^. Moreover, the prevalence of LC increases from the time of IPF diagnosis^5,6^. Ozawa *et al*. reported that the cumulative prevalence of LC is increased to 3.3%, 15.4%, and 54.7% after 1, 5, and 10 years from IPF diagnosis^7^. Previous studies showed that the prevalence of LC and IPF (LC-IPF) is higher in older men, those who smoke, and those with squamous cell carcinoma^8,9^. Although the exact relationship between LC and IPF has not yet been established, epigenetic changes, genetic changes, and oxidative stress are thought to be involved in the development of LC-IPF^10^.

In the treatment of patients with LC-IPF, physicians are reluctant to treat LC because of the poor prognosis of IPF^11^. In addition, complications such as pneumonia, acute exacerbation of IPF (AE-IPF), acute respiratory distress syndrome, and air leakage after lung surgery are relatively more frequent in LC-IPF patients than in those with LC alone, thus resulting in higher mortality rates^12,13^. These complications and high mortality makes the treatment of LC difficult. Although some studies suggest that surgical treatment is effective in IPF patients with early stage LC, little is known about the treatment, prognosis, and long-term survival of advanced stage patients^11,14,15^.

In 2012, Ley *et al*.^16^ reported the GAP index and staging system, which can be easily used to predict the mortality and the timing of lung transplantation in IPF patients. Because the GAP index and stage can be obtained simply using gender (G), age (A), and two lung physiologic variables (P) (FVC and DL_CO_), clinicians can calculate the GAP score easily.

In this study, we aimed to investigate the effectiveness of the treatment of LC and the prognosis in patients with LC-IPF according to disease severity and treatment modality.

## Results

### Patients’ baseline characteristics

Overall, 160 patients with LC-IPF were divided into GAP stage I (n = 115), II (n = 36), and III (n = 9), and each GAP stage was classified into LC clinical stage I (n = 45), II (n = 23), III (n = 45), and IV (n = 47). Because of the few patients in GAP stage III (n = 9), GAP stage III was combined with GAP stage II.

Table 1 shows the comparison of the patients’ baseline characteristics according to the GAP stage. The mean patient age was 70.1 ± 8.2 years, and majority of the patients were men (94.4%). The median OS was 17.7 months. The age, gender, body mass index, and total amount of cigarettes smoked in a lifetime among patients in GAP stage 1 were not significantly different from those in GAP stages II and III. Patients with high Eastern Cooperative Oncology Group (ECOG) score and advanced LC were significantly more distributed in the higher GAP stages than in the GAP stage I (*p* < 0.001 and *p* < 0.001, respectively). Moreover, lung function, median overall survival (OS), and median progression-free survival (PFS) were lower in high GAP stage than in low GAP stage (*p* < 0.001, *p* < 0.001, and *p* < 0.001, respectively). In terms of histologic type, the distribution of squamous cell carcinoma and adenocarcinoma was similar in GAP stage I.

**Table 1:** Baseline characteristics according to GAP stage.

	Total (n = 160)	GAP I (n = 115)	GAP II/III (n = 45)	*p* value
Age (year)	70.1 ± 8.2	69.6 ± 8.9	71.5 ± 6.0	0.200
Gender (male)	151 (94.4)	107 (93.0)	44 (97.8)	0.243
BMI, kg/m²	23.0 ± 3.3	23.2 ± 2.9	22.5 ± 4.0	0.191
Smoking status				0.763
Never smoker	15 (9.4)	12 (10.4)	3 (6.7)	
Former smoker	114 (71.3)	81 (70.4)	33 (73.3)	
Current smoker	31 (19.4)	22 (19.1)	9 (20.0)	
Total amount of cigarettes smoked in a lifetime (PYs)	37.4 ± 25.7	35.0 ± 22.5	43.8 ± 31.8	0.125
Pulmonary function test				
FVC, predicted %	85.6 ± 18.3	92.5 ± 14.6	68.0 ± 14.6	<0.001
FEV_1_, predicted %	89.9 ± 19.6	96.3 ± 17.1	73.6 ± 15.8	<0.001
DLco, predicted %	73.4 ± 20.7	79.1 ± 17.7	57.5 ± 20.5	<0.001
Histology				0.004
Squamous cell carcinoma	76 (47.5)	52 (45.2)	24 (53.3)	
Adenocarcinoma	60 (37.5)	49 (42.6)	11 (24.4)	
Large-cell carcinoma	4 (2.5)	4 (3.5)	0	
Unclassified NSCLC	20 (12.5)	10 (8.7)	10 (22.2)	
Location of lung cancer				0.550
Right upper lobe	30 (18.7)	21 (18.3)	9 (20.0)	
Right middle lobe	6 (3.8)	5 (4.3)	1 (2.2)	
Right lower lobe	59 (36.9)	40 (34.8)	19 (42.2)	
Left upper lobe	30 (18.7)	24 (20.9)	6 (13.3)	
Left lower lobe	35 (21.9)	25 (21.7)	10 (22.2)	
Clinical lung cancer stage				<0.001
I/II/III/IV	45/23/45/47	39/20/35/21	6/3/10/26	
ECOG				<0.001
0/1/2/3/4	29/86/28/12/5	26/71/11/5/2	3/15/17/7/3	
Primary treatment				<0.001
Conservative care	22 (13.8)	11 (9.6)	11 (24.4)	
Surgery	69 (43.1)	62 (53.9)	7 (15.6)	
Chemotherapy	58 (36.3)	35 (30.4)	23 (51.1)	
Radiotherapy	11 (6.9)	7 (6.1)	4 (8.9)	
Median OS, month	17.7	22.4	6.7	<0.001
Median PFS, month	7	10.5	3.9	<0.001
Overall mortality	111 (69.4)	74 (64.3)	37 (82.2)	0.027

Abbreviations: GAP = gender (G), age (A), and two physiology variables (P) (FVC and DLco) stage system; BMI = body mass index; PYs = pack-years; FVC = forced vital capacity; FEV_1_ = forced expiratory volume in one second; DLco = diffusing capacity for carbon monoxide; NSCLC = non-small-cell lung cancer; ECOG = Eastern Cooperative Oncology Group; OS = overall survival; PFS = progression-free survival.

Data are presented as mean ± standard deviation, median, or frequency (%).

### Patients’ baseline characteristics according to treatment modality

The 115 patients with GAP stage I were divided according to the treatment modality. Table 2 shows the baseline characteristics. Of these patients, the mean age was the highest in among patients who received conservative care (77.8 ± 6.1, *p* = 0.006); the number of patients with advanced LC stage and those with poor performance status (PS) were significantly higher among those who received chemotherapy and radiotherapy (*p* < 0.001 and *p* < 0.001, respectively). Patients with GAP stage I who underwent surgery showed a significantly longer median PFS and OS than those who received other treatment modalities.

**Table 2:** Comparison of characteristics according to treatment modality in GAP stage I.

	Conservative care (n = 11)	Operation (n = 62)	Chemotherapy (n = 35)	Radiotherapy (n = 7)	*P* value
Age	77.8 ± 6.1	69.4 ± 8.4	67.2 ± 9.5	70.3 ± 7.9	0.006
Gender (male)	9 (81.8)	60 (96.8)	32 (91.4)	6 (85.7)	0.289
BMI, kg/m²	22.6 ± 2.6	23.6 ± 3.1	22.9 ± 2.8	22.2 ± 2.1	0.433
Smoking status					0.135
Never smoker	0	8 (12.9)	4 (11.4)	0	
Former smoker	8 (72.7)	47 (75.8)	22 (62.9)	4 (57.1)	
Current smoker	3 (27.3)	7 (11.3)	9 (25.7)	3 (42.9)	
Total amount of cigarettes smoked in a lifetime (PYs)	50.9 ± 20.8	32.1 ± 21.3	35.1 ± 24.9	34.3 ± 15.7	0.095
Pulmonary function test					
FVC, predicted %	98.6 ± 15.7	95.2 ± 13.5	86.9 ± 14.5	86.9 ± 15.6	0.068
FEV_1_, predicted %	106.6 ± 14.5	97.2 ± 16.7	93.2 ± 17.6	87.3 ± 16.6	0.078
DL_CO_, predicted %	75.2 ± 18.8	81.7 ± 16.3	78.4 ± 19.6	65.4 ± 14.0	0.109
Histologic type					0.009
Squamous cell carcinoma	6 (54.5)	30 (48.4)	13 (37.1)	3 (42.9)	
Adenocarcinoma	4 (36.4)	28 (45.2)	16 (45.7)	1 (14.3)	
Large cell carcinoma	0	4 (6.5)	0	0	
Unclassified NSCLC	1 (9.1)	0 (0)	6 (17.1)	2 (42.9)	
Clinical stage					<0.001
I/II/III/IV	4/2/4/1	33/17/11/1	0/0/18/17	2/1/2/2	
ECOG					<0.001
0/1/2/3/4	1/7/2/0/1	22/37/3/0/0	3/25/5/2/0	0/2/1/3/1	
Median OS, month	13.4 ± 9.4	42.0 ± 37.5	11.2 ± 18.4	11.9 ± 7.8	<0.001
Median PFS, month	3.9 ± 4.0	34.6 ± 39.0	7.5 ± 18.3	8.2 ± 6.1	<0.001

Abbreviations: GAP = gender (G), age (A), and two physiology variables (P) (FVC and DLco)) stage system; BMI = body mass index; PYs = pack-years; FVC = forced vital capacity; FEV_1_ = forced expiratory volume in one second; DLco = diffusing capacity of the lung for carbon monoxide; NSCLC = non-small-cell lung cancer; ECOG = Eastern Cooperative Oncology Group; OS = overall survival; PFS = progression-free survival.

Data are presented as mean ± standard deviation, median, or frequency (%).

The age, gender, total amount of cigarettes smoked, and PFT result according to treatment modality were not significantly different between GAP stage II and III (Table 3). Similar to those with GAP stage I, the number of patients with early LC was higher in the surgery group than in the other treatment groups. Among the treatment modalities, OS and PFS were the longest in surgery, but the OS was considerably lower for of patients with GAP stages II and III than for those with GAP stage I.

**Table 3:** Comparison of characteristics according to treatment modality in GAP stage II and III.

	Conservative care (n = 11)	Operation (n = 7)	Chemotherapy (n = 23)	Radiotherapy (n = 4)	*P* value
Age (years)	73.1 ± 7.2	71.4 ± 7.3	71.0 ± 5.4	70.3 ± 5.5	0.925
Gender (male)	10 (90.9)	7 (100.0)	23 (100.0)	4 (100.0)	0.409
BMI, kg/m²	22.6 ± 4.7	25.7 ± 3.8	21.4 ± 3.5	22.9 ± 3.8	0.132
Smoking status					0.083
Never smoker	0	0	3 (13.0)	0	
Former smoker	10 (90.9)	7 (100.0)	14 (60.9)	2 (50.0)	
Current smoker	1 (9.1)	0	6 (26.1)	2 (50.0)	
Total amount of cigarettes smoked in a lifetime (PYs)	40.1 ± 20.4	36.4 ± 21.4	39.1 ± 27.1	93.8 ± 58.5	0.087
Pulmonary function test					
FVC, predicted %	70.7 ± 16.0	72.9 ± 5.3	64.3 ± 16.2	72.8 ± 8.5	0.436
FEV_1_, predicted %	75.6 ± 19.7	78.6 ± 5.2	70.0 ± 16.5	79.3 ± 10.1	0.496
DL_CO_, predicted %	56.5 ± 17.9	67.7 ± 25.1	55.6 ± 20.3	51.7 ± 18.6	0.610
Histologic type					0.113
Squamous cell carcinoma	8 (72.7)	6 (85.7)	9 (39.1)	1 (25.0)	
Adenocarcinoma	2 (18.2)	1 (14.3)	7 (30.4)	1 (25.0)	
Unclassified NSCLC	1 (9.1)	0	7 (30.4)	2 (50.0)	
Clinical stage					<0.001
I/II/III/IV	1/1/1/8	4/2/0/1	0/0/9/14	1/0/0/3	
ECOG					0.260
0/1/2/3/4	0/3/4/2/2	0/4/3/0/0	3/7/9/4/0	0/1/1/1/1	
Median OS, month	4.5	24.5	6.9	9.0	0.011
Median PFS, month	1.8	12.5	3.6	5.0	0.003

Abbreviations: GAP = gender (G), age (A), and two physiology variables (P) (FVC and DLco)) stage system; BMI = body mass index; PYs = pack-years; FVC = forced vital capacity; FEV_1 =_ forced expiratory volume in one second; DLco = diffusing capacity of the lung for carbon monoxide; NSCLC = non-small-cell lung cancer; ECOG = Eastern Cooperative Oncology Group; OS = overall survival; PFS = progression-free survival.

Data are presented as mean ± standard deviation, median, or frequency (%).

### Survival analysis

**Figure 1:**
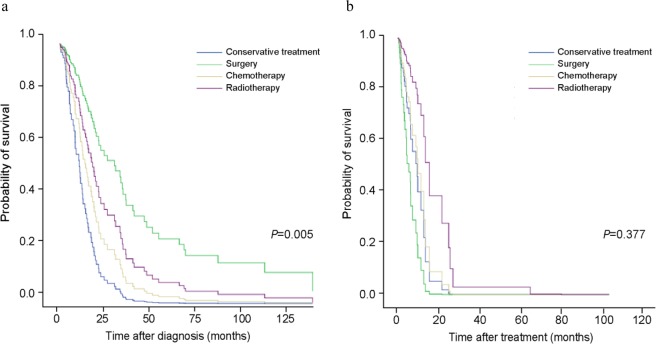
Comparison of survival probability according to treatment modality in GAP stage I (**a**) and GAP ((gender [G], age [A]), and two physiology variables (P) (FVC and DL_CO_)) stage system) stage II and III (**b**). Cox regression models were adjusted for lung cancer clinical stages, Eastern Cooperative Oncology Group (ECOG), primary treatment, and total amount of cigarettes smoked in a lifetime.

In the survival analysis of all patients, smoking status, ECOG PS, GAP stage, primary treatment modality, and LC clinical stage were adjusted for using Cox proportional hazard regression model. Age, gender, and PFT were not included in the Cox proportional hazards model because they are variables of the GAP staging system. Table 4 shows the survival analysis according to the GAP stage. In GAP stage I, the most significant increase in survival probability was in the surgery group (Fig. 1a; hazard ratio [HR] = 0.233; 95% confidence interval [CI]: 0.094–0.574; *p* = 0.005). However, unlike GAP stage I, no survival benefit was found in the surgery group as in the GAP stages II and III (Fig. 1b; *p* = 0.377). Advanced LC stage and higher ECOG PS were substantially associated with poor outcome. Smoking status was a significant prognostic factor in GAP stages II and III (*p* = 0.031).

**Table 4:** Cox multivariate proportional hazard analysis according to GAP stage.

Variables	GAP stage I			GAP stage II/III		
	HR	95% CI	*P* value	HR	95% CI	*P* value
Lung cancer stage			0.001			0.002
Stage I	1.000			1.000		
Stage II	1.489	0.722–3.072	0.281	0.183	0.023–1.455	0.108
Stage III	1.508	0.715–3.179	0.281	5.384	0.520–55.729	0.158
Stage IV	7.775	2.593–23.307	<0.001	35.94	3.606–358.215	0.002
ECOG			0.077			0.007
ECOG 0 and 1	1.000			1.000		
ECOG 2	1.134	0.471–2.732	0.779	3.71	1.436–9.587	0.007
ECOG 3 and 4	3.037	1.152–8.010	0.025	4.711	1.558–14.240	0.006
Primary treatment			0.005			0.377
Conservative care	1.000			1.000		
Operation	0.233	0.094–0.574	0.002	2.118	0.261–17.207	0.482
Chemotherapy	0.613	0.210–1.787	0.370	0.818	0.296–2.265	0.700
Radiotherapy	0.421	0.115–1.541	0.191	0.323	0.077–0.077	0.123
Total amount of cigarettes smoked in a lifetime (PYs)	0.993	0.982–1.004	0.189	1.016	1.002–1.031	0.031

Abbreviations: HR = Hazard ratio, GAP = gender (G), age (A), and two physiology variables (P) (FVC and DLco)) stage system; PYs = pack-years; ECOG = Eastern Cooperative Oncology Group.

*Adjusted for lung cancer clinical stage, ECOG, primary treatment, and smoking amount.

**Figure 2:**
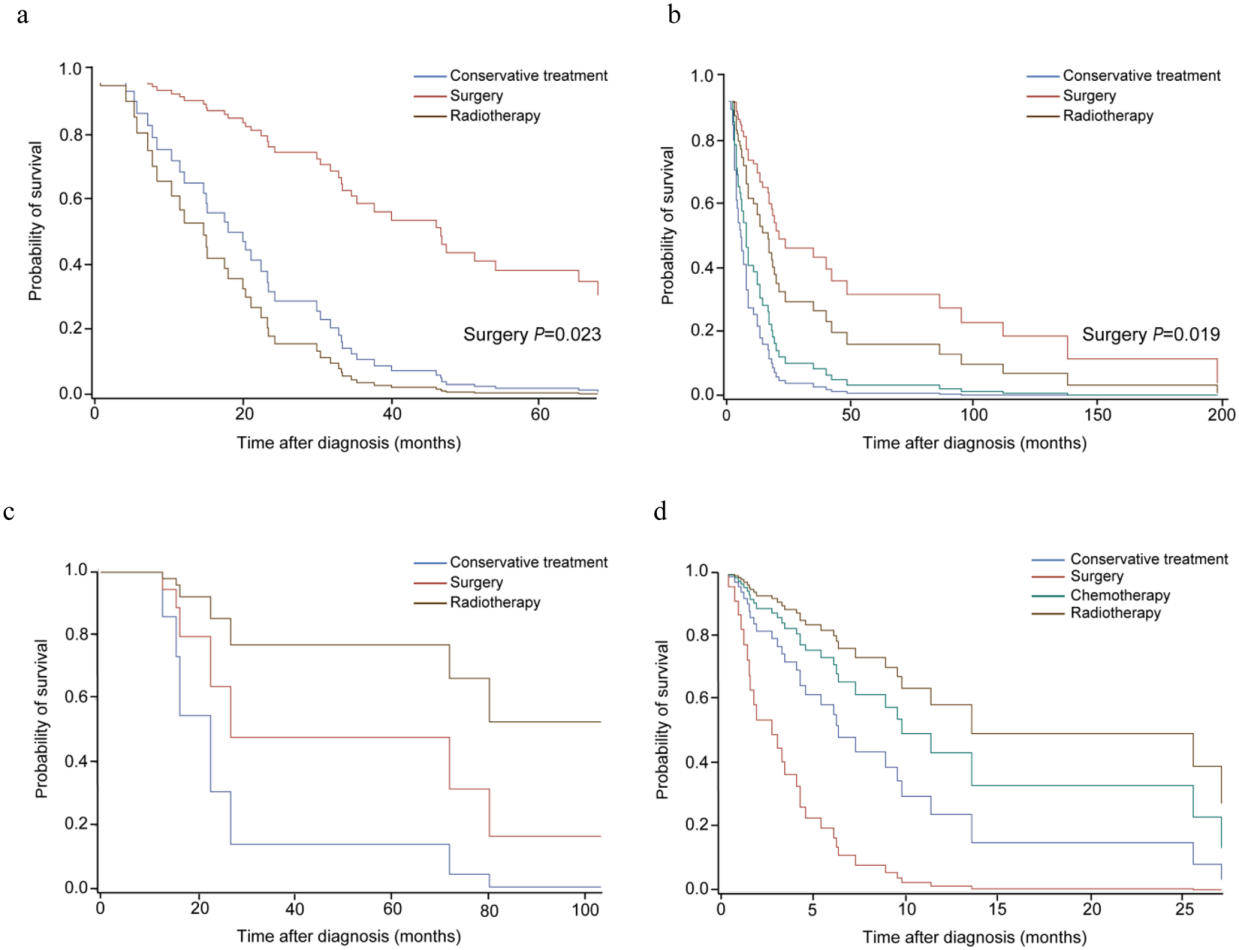
Subgroup analysis for survival according to the primary treatment after classifying the patient as GAP ((gender [G], age [A]), and two physiology variables (P) (FVC and DL_CO_)) stage system) stage and the lung cancer (LC) stage. (**a**) GAP stage I and LC stages I and II, (**b**) GAP stage I and LC stage III and IV, (**c**) GAP stage II and III and LC stage I and II, and (**d**) GAP stages II and III and LC stage III and IV. In GAP stage I, surgery significantly improved the survival in both early and advanced LC stages (*p* = 0.023 and *p* = 0.019). In GAP stages II and III, any treatment modalities failed to significantly improve survival in early or advanced LC stages.

Each GAP stage was also divided into early LC and advanced LC (Fig. 2 and Supplementary Table S1). In both early and advanced LC stages with GAP stage I, survival was significantly increased in the surgery group (*p* = 0.020 in GAP stage I and *p* = 0.020 in GAP stage II/III). In GAP stages II and III, any treatment modalities failed to considerably improve survival in early or advanced LC stages. ECOG PS was significantly correlated with mortality in advanced LC.

### Classification according to the GAP stage and treatment modality

Of the 160 patients, 22, 69, 58, and 11 received conservative therapy, surgery, chemotherapy, and radiation therapy as primary treatment, respectively. AE-IPF occurred in 1 (4.5%), 10 (14.5%), 7 (12.1%), and 4 (36.4%) patients who received conservative therapy, surgery, chemotherapy, and radiotherapy, respectively (Table 5). The toxicity of all AE was higher than grade 4. AE-IPF was significantly more frequent in GAP stage II/III than in GAP stage I in patients who received both surgery and chemotherapy (*p* = 0.050 and *p* = 0.007, respectively). In particular, in patients who received chemotherapy, mortality owing to AE was also significantly higher in GAP stage II/III than in GAP stage I (*p* = 0.002). Most complications such as prolonged air leakage, subcutaneous emphysema, cytopenia, or pneumonia other than AE were lower than grade 3 and did not differ according to the GAP stages.

**Table 5:** Complications that occurred after treatment classified according to GAP stage and treatment modality.

	GAP stage I (n = 115)	GAP stage II/III (n = 45)	*p* value
Operation (n = 69) (GAP stage I (n = 62), GAP stage II/III (n = 7)			
Prolonged air leak	9 (14.5)	—	0.150
Subcutaneous emphysema	4 (6.5)	—	0.347
Pneumonia	1 (1.6)	2 (28.6)	0.014
Pneumothorax	6 (9.7)	2 (28.6)	0.191
AE-IPF	7 (11.3)	3 (42.9)	0.050
Others	3 (4.9)	—	0.414
Chemotherapy (n = 58) (GAP stage I (n = 35), GAP stage II/III (n = 23)			
Cytopenia	3 (8.6)	2 (8.7)	0.987
GI trouble	3 (8.6)	1 (4.3)	0.523
Skin eruption	1 (2.9)	1 (4.3)	0.763
Pneumonia	1 (2.9)	1 (4.3)	0.763
Septic shock	—	1 (4.3)	0.175
Pneumonitis	2 (6.9)	—	0.142
AE-IPF	1 (2.9)	6 (26.1)	0.007
Radiotherapy (n = 11) (GAP stage I (n = 7), GAP stage II/III (n = 4)			
Pneumonia	1 (14.3)	—	0.227
AE-IPF	2 (28.6)	2 (50.0)	0.480

Abbreviations: GAP = gender (G), age (A), and two physiology variables (P) (FVC and DLco)) stage system; GI = gastro-intestinal, AE-IPF = acute exacerbation of idiopathic pulmonary fibrosis.

The AE-IPF was defined within one month after surgical resection in surgery, while it was defined as the onset of AE-IPF within one month after one cycle in chemotherapy and radiotherapy.

Other complications in surgery included one case each of acute thrombosis, post-operation atrial fibrillation, and post-operation atelectasis.

Note: Values in parentheses are percentages.

## Discussion

The standard treatment modality for LC-IPF is yet to be established because of the short median OS due to IPF, the high complication rate occurring after anti-cancer treatment such as AE-IPF, and the rarity of comorbidity of IPF and LC^17,18^. In this study, we investigated the efficiency of treatment and prognosis of LC-IPF according to treatment modalities considering the severity of LC and IPF, ECOG PS, and smoking status, while previous studies primarily focused on the treatment of LC stage. Our study showed that surgery was an effective treatment modality for GAP stage I patients with LC, while other treatment modalities of LC failed to show effectiveness regardless of the GAP stage.

In 2015, Tomassetti *et al*.^15^ described that LC developing in IPF significantly decreased the median OS compared with IPF without LC (LC-IPF: 38.7 months, IPF without LC: 63.9 months; HR = 5.0; 95% CI: 21.91–8.57; *p* < 0.001). Saito *et al*.^19^ reported the survival rates after pulmonary resection of 350 patients with pathologic stage IA and found that the 5-year survival rate was significantly reduced in patients with IPF (54.2% in LC-IPF and 88.3% in LC only, *p* < 0.001), and IPF was the only prognostic factor (*p* = 0.04). These studies showed that compared with LC alone or IPF alone, LC-IPF is related with poor prognosis. In our study, the prognosis of LC-IPF was also poor; the median OS and PFS were 17.7 and 7.0 months, respectively. Additionally, as the GAP stage increased, the survival of patients with LC-IPF rapidly reduced in all treatment groups compared with that in GAP stage I. This could explain why there is no effective treatment modality in higher GAP stages. It is possible that low efficacy of LC treatments because of poor survival is likely to be more affected by the progression and severity of IPF itself than by LC severity. Watanabe *et al*. reported that surgery for LC can be helpful in selective patients with LC-IPF. In their study, although the hospital mortality (7.1% vs 1.9%, *p* = 0.03) or AE (7.1% vs 0.0%, *p* < 0.001) rate after lung surgery was significantly higher in patients with LC-IPF than in LC only patients, the 5-year survival rate for stage I LC with IPF was 61.6% compared with 83.0% in patients without IPF. Although some studies reported that the history of AE, surgical procedure, male sex, elevated LDH/KL-6, preoperative steroid use, and low FVC are risk factors for mortality or AE of interstitial pneumonia, these studies were performed with relatively small numbers of patients than those in our study and did not suggest a clear selection criteria of patients for whom surgery could be beneficial^12,20–22^. In the current study, surgery was an effective treatment modality in LC patients with GAP stage I. This finding indicates that the assessment of IPF according to the GAP stage could be helpful in identifying patients who would benefit from surgery. Furthermore, our study showed that advanced LC stage and poor PS were significantly related with poor outcomes. However, the high frequency of AE and AE-related mortality associated with anticancer therapy is an important issue for determining the need for surgery even at low LC stages. The incidence rate of AE ranges from 7.1% to 33.0% in surgery^14,23,24^. In the present study, the incidence of AE was 14.5% in the surgery group. Moreover, the incidence of AE was significantly higher in patients with advanced GAP stage. As such, efforts are being made to prevent the development of AE. A recent study reported that perioperative pirfenidone significantly decreased the incidence of AE compared with control, although this was a retrospective study and included only a small number of patients^25^. Therefore, physicians should be cautious regarding the occurrence of AE when performing surgery in LC patients with advanced GAP stage and should consider the use of pirfenidone during the perioperative period.

The clinical value of chemotherapy and radiotherapy has been studied in patients with inoperable conditions; however, their role remains unclear owing to the significantly lower OS and higher complication rates than those for surgery. Chen *et al*. performed a meta-analysis to investigate the efficacy and safety of chemotherapy in patients with non-small-cell LC (NSCLC) and interstitial lung disease (ILD)^26^. In their study, although chemotherapy was associated with a higher incidence of AE, high overall response rate (ORR) and disease control rate (DCR) were achieved (41.3% and 77.7%, respectively), and the 1-year survival rate was approximately 29.4%. Watanabe *et al*.^27^ reported the efficacy of chemotherapy in 23 patients with LC-IPF. In their study, the ORR, DCR, and 1-year survival rate were 42.9%, 81.0%, and 28.6%. As such, they concluded that chemotherapy might be effective in NSCLC patients with ILD or IPF. However, these studies reached their conclusions either indirectly through comparison with other studies or by conducting a study with only a small number of patients. In contrast, Kanaji *et al*.^28^ reported a median PFS and median OS after chemotherapy that were significantly shorter in LC-IPF patients than in LC patients without ILD. In their study, DCR was also significantly lower in patients with LC-IPF than in those with NSCLC alone (87% for non-ILD and 53% for IPF, *p* < 0.001). Furthermore, some studies reported incidence rates of AE ranging from 5.6% to 30% with chemotherapy^29,30^. Kato *et al*. showed that pemetrexed-related pulmonary toxicity developed in 12.0% of patients with IIP and in 1.1% of patients without IIP, with a significantly higher proportion of patients with IIP (odds ratio: 11.8; *p* = 0.03)^31^. According to a study by Kenmotsu *et al*.^30^, patients with a UIP pattern had a significantly higher incidence of chemotherapy-associated AE at 30% than patients with a non-UIP pattern at 8% (*p* = 0.005). Furthermore, AE of grade 3 or higher occurred in a higher proportion of patients with UIP pattern (*p* = 0.003). In our study, although it was not significant, chemotherapy tended to decrease the risk of mortality. Additionally, AE was significantly more frequent in patients with advanced GAP stage in the chemotherapy group (*p* = 0.007). Therefore, when deciding the applicability of chemotherapy in inoperable cases, the GAP stage might be a helpful criterion. Targeted therapy for LC has recently been introduced, and immune therapy has also attracted attention. Further large studies are needed to investigate the efficacy or safety of chemotherapy in LC-IPF.

Few studies on the effect of radiotherapy in LC-IPF have been conducted. Although radiotherapy is as effective as surgery in patients with early LC without IPF, it yields poor outcomes in LC-IPF^32–34^. Some researchers speculate that the incidence of acute respiratory deterioration will increase in radiotherapy; however, little research has been conducted to support this theory. In a study of curative radiotherapy in 7 patients with IPF, 2 patients showed partial remission and 5 patients did not achieve such change. Moreover, acute respiratory deterioration occurred in 5 patients^14^. According to Bahig *et al*.^34^, of the 150 patients with stage I NSCLC in their study, 5 patients had features of IPF, of which three developed grade 5 pneumonitis. Collectively, the above studies indicate that patients with LC-IPF have a higher risk of severe toxicity. Similar to previous studies, 4 of the 11 patients experienced AE, and the incidence of AE tended to increase as the GAP stage advanced in our study. This result suggested that radiotherapy had limited therapeutic benefit in LC-IPF, and the applicability of radiotherapy should be decided by a multidisciplinary team of pulmonologist, radiologist, clinical oncologist, and radiation oncologist.

There are several limitations in this study. First, in the present study, IPF diagnosis was performed by chest CT without tissue analysis in a relatively large number of patients (n = 130, 81.2%). However, as highlighted in the 2018 Guidelines for accurate diagnosis, the multidisciplinary approach, including pulmonologists, chest specific radiologists, and a pulmonary pathologist, increased the accuracy of the diagnosis. Second, the conservative care group might have individual characteristics that may make them ineligible for anticancer therapies. However, the ECOG PS and clinical stage of LC in the conservative care group were not worse than those in other treatment groups, and the GAP stage and smoking amount were considered in this study. The comorbidity was also not different from other treatment groups (data not shown). Third, there were only few GAP stage III patients. When calculating the GAP stage, patients who failed to perform DL_CO_ owing to respiratory insufficiency might have been given a point in the “DL_CO_ cannot perform” category, and we could not verify the data of those patients due to the retrospective study design. There is need for further studies on GAP stage III patients. Finally, because radiotherapy tends to be performed selectively in inoperable cases, only a small number of patients received radiotherapy as primary treatment.

In conclusion, active therapies such as surgery in LC-IPF patients with GAP stage I are recommended. However, at the advanced GAP stage, no beneficial treatment modalities were found. Therefore, physicians should carefully evaluate the patient’s condition, and the applicability of surgery in patients with advanced LC and higher GAP stages should be decided by a multidisciplinary team.

## Methods

### Study population

**Figure 3:**
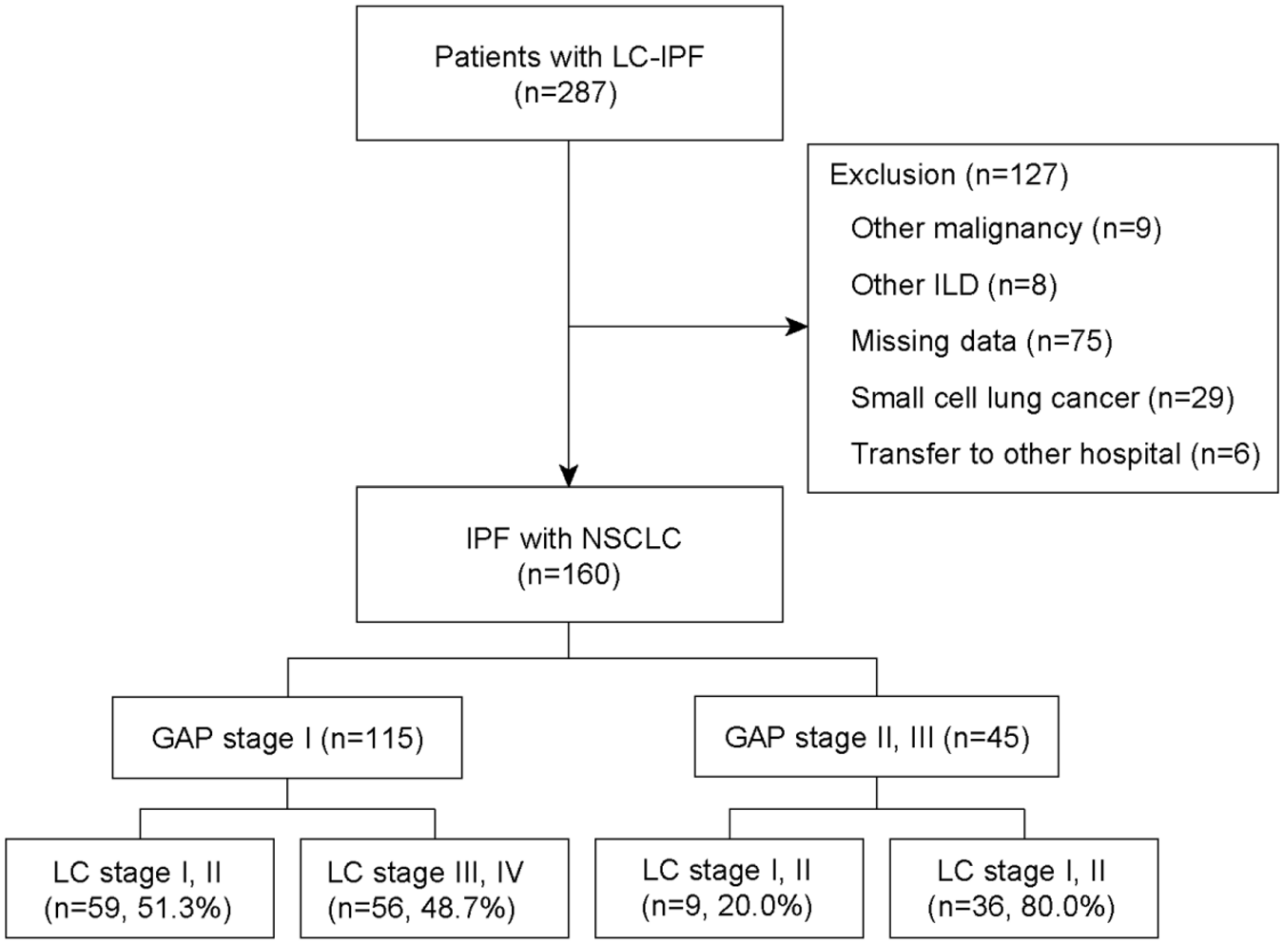
Patient recruitment flow chart. Abbreviation: LC-IPF = lung cancer with idiopathic pulmonary fibrosis; ILD = interstitial lung disease; ECOG = Eastern Cooperative Oncology Group; GAP = gender (G), age (A), and two physiology variables (P) (FVC and DL_CO_) stage system.

The study was conducted retrospectively in Seoul National University Bundang Hospital, from 1 November 2003 to 31 August 2016. Initially, 287 patients with LC-IPF were enrolled, and IPF was diagnosed and confirmed by a multidisciplinary team consisting of pulmonologists, a chest specific radiologist, and a pulmonary pathologist in accordance with the 2011 diagnostic criteria set by the International Consensus Statement of the American Thoracic Society and European Respiratory Society^1^. Only patients with biopsy-confirmed LC were included. Of the 287 patients, 127 were excluded due to malignancy other than LC (n = 9), IIP other than IPF (n = 8), incomplete data such as no PFT results or non-biopsy proven LC (n = 75), small-cell LC (n = 29), and transfer to other hospital without treatment (n = 6). Finally, 160 patients with LC-IPF were examined. GAP stage I comprised the most patients (n = 115), followed by GAP stage II and III at 35 and 9, respectively. The patient enrolment flow chart is shown in Fig. 3. The Institutional Review board and Ethics Committee of Seoul National University Bundang Hospital approved the study (IRB number: B-1707/411-402). Written informed consent was waived owing to the retrospective nature of the study. All methods were performed in accordance with the Declaration of Helsinki.

### Data collection and statistical analysis

The age, gender, smoking history, comorbidity, ECOG PS at treatment initiation, and time of LC diagnosis and recurrence were investigated in this study using data from the patient’s medical records. The PFT results were used within one month of LC diagnosis. All data represented as mean ± S.D. unless otherwise stated. High-resolution CT, positron emission tomography, brain magnetic resonance imaging, and bronchoscopy confirmed the location, clinical stage, and fibrotic area involvement of LC. LC was pathologically confirmed via bronchoscopic biopsy, percutaneous needle aspiration, or surgical lung biopsy. Early LC was defined as stage I or II, while advanced LC was defined as stage III or IV. The GAP score was calculated using gender (0–2 points), FVC (4–5 points), and DL_CO_ (0–3 points), and classified into stages I (0–3 points), II (4–5 points), or III (6–8 points)^16^. AE-IPF was defined as that occurring within 4 weeks after surgical resection and within 4 weeks after the first cycle of chemotherapy or radiotherapy^17,18^. PFS was calculated from the beginning of the treatment to the development of recurrence, whereas OS was calculated starting from the diagnosis of LC-IPF to the date of death or last follow-up.

### Statistical analysis

Categorical variables were analysed using Chi-squared distribution or Fisher’s exact test, and continuous variables were analysed using the Student t-test or Mann-Whitney U test. Cox proportional hazard regression analysis and Firth logistic regression were used to identify the risk factors of survival of LC-IPF patients. Survival time was calculated starting from the date of LC diagnosis to the date of death or last follow-up. Survival was investigated using data from the Ministry of Public Administration and Security and medical charts between 2003 and 2017. All statistical analyses were performed using IBM SPSS Statistics (version 22.0). An adjusted p-value of < 0.05 was considered significant in this study.

## Supplementary information

Supplementary Table S1

## Data Availability

The datasets generated during the current study are available from the corresponding author on request.
