# Supplementary material for: Prognosis of non-small-cell lung cancer in patients with idiopathic pulmonary fibrosis
Source: Sci Rep. 2019 Aug 29;9:12561. doi: 10.1038/s41598-019-49026-y (PMC6715712; doi:10.1038/s41598-019-49026-y)
Supplement: Supplementary file 1 — Supplementary Table S1 [file 41598_2019_49026_MOESM1_ESM.docx]

**Prognosis of non-small-cell lung cancer in patients with idiopathic pulmonary fibrosis**

SongYi Han^1,2^, Yeon Joo Lee^1^, Jong Sun Park^1^, Young-Jae Cho^1^, Ho Il Yoon^1^, Jae-Ho Lee^1^, Choon-Taek Lee^1^, Jin-Haeng Chung^3^, Kyung Won Lee^4^, Sang Hoon Lee*^5^

^1^Division of Pulmonary and Critical Care Medicine, Department of Internal Medicine, Seoul National University Bundang Hospital, 82 Gumi-ro, 173 Beon-gil, Bundang-gu, Seongnam-si, Gyeonggi-do, 463-707, Republic of Korea

^2^Division of Hospital Medicine, Department of Internal Medicine, Yonsei University College of Medicine, Yonsei University Health System 50-1 Yonsei-ro, Seodaemun-gu, Seoul 120-752, Korea

^3^Department of Pathology, Seoul National University Bundang Hospital, 82 Gumi-ro, 173 Beon-gil, Bundang-gu, Seongnam-si, Gyeonggi-do, 463-707, Republic of Korea

^4^Department of Radiology, Seoul National University Bundang Hospital, 82 Gumi-ro, 173 Beon-gil, Bundang-gu, Seongnam-si, Gyeonggi-do, 463-707, Republic of Korea

^5^Division of Pulmonary and Critical Care Medicine, Department of Internal Medicine, Institute of Chest Diseases, Severance Hospital, Yonsei University College of Medicine. 50-1 Yonsei-ro, Seodaemun-gu, Seoul 120-752, Korea

***Corresponding Author:** Sang Hoon Lee, M.D.

Division of Pulmonary and Critical Care Medicine, Department of Internal Medicine

Yonsei University College of Medicine, Yonsei University Health System 50-1 Yonsei-ro, Seodaemun-gu, Seoul 120-752, Korea

Tel: +82.2-2228-1955

Fax: +82.2-393-6884

E-mail address: [cloud9](mailto:tearpoem9@gmail.com)@yuhs.ac

**Supplementary Table S1.** Analysis of survival probability according to GAP stage and lung cancer stage

|  |  | Lung cancer clinical stage I/ II | | | Lung cancer clinical stage III/ IV | | |
| --- | --- | --- | --- | --- | --- | --- | --- |
|  | Variable | HR | 95% CI | *P* value | HR | 95% CI | *P* value |
| GAP stage I | ECOG |  |  |  |  |  |  |
|  | ECOG 0 and 1 | 1.000 |  |  | 1.000 |  |  |
|  | ECOG 2 | 2.359 | 0.762–7.303 | 0.137 | 0.804 | 0.313–2.066 | 0.650 |
|  | ECOG 3 and 4 | 2.077 | 0.380–11.365 | 0.399 | 3.677 | 1.170–11.549 | 0.026 |
|  | Primary treatment |  |  |  |  |  |  |
|  | Conservative care | 1.000 |  |  | 1.000 |  |  |
|  | Operation | 0.237 | 0.069–0.817 | 0.023 | 0.232 | 0.068–0.786 | 0.019 |
|  | Chemotherapy |  |  |  | 0.690 | 0.245–1.942 | 0.483 |
|  | Radiotherapy | 1.490 | 0.306–7.303 | 0.621 | 0.368 | 0.068–1.991 | 0.246 |
|  | Total amount of cigarettes smoked for life (PYs) | 0.989 | 0.971–1.006 | 0.205 | 0.995 | 0.983–1.008 | 0.463 |
| GAP stage II/III | ECOG |  |  |  |  |  |  |
|  | ECOG 0 and 1 | 1.000 |  |  | 1.000 |  |  |
|  | ECOG 2 | 1.463 | 0.263–8.144 | 0.664 | 2.200 | 0.839–5.767 | 0.109 |
|  | ECOG 3 and 4 |  |  |  | 4.771 | 1.443–15.777 | 0.011 |
|  | Primary treatment |  |  |  |  |  |  |
|  | Conservative care | 1.000 |  |  | 1.000 |  |  |
|  | Operation | 0.377 | 0.025–5.796 | 0.484 | 3.041 | 0.375–24.673 | 0.298 |
|  | Chemotherapy |  |  |  | 0.582 | 0.209–1.622 | 0.301 |
|  | Radiotherapy | 0.134 | 0.000–1380.664 | 0.670 | 0.374 | 0.083–1.678 | 0.199 |
|  | Total amount of cigarettes smoked for life (PYs) | 1.025 | 0.965–1.088 | 0.424 | 1.005 | 0.990–1.021 | 0.506 |

Abbreviations: HR = Hazard ratio; CI, confidence interval; GAP = gender (G), age (A), and two physiology variables (P) (FVC and DLco) stage system; PYs = pack-years; ECOG = Eastern Cooperative Oncology Group

*Adjusted for ECOG, primary treatment, and smoking amount
